# Clinical effect of intravascular interventional therapy in the treatment of acute ischemic stroke and its influence on cognitive function, cerebral hemodynamics and inflammatory factors

**DOI:** 10.12669/pjms.38.5.5255

**Published:** 2022

**Authors:** Kai-long Liu, Jie He, Ya-jing Zang

**Affiliations:** 1 Kai-long Liu, Department of Neurology, Tangxian people’s Hospital, Baoding 072350, Hebei, China; 2 Jie He, Department of Neurology, Tangxian people’s Hospital, Baoding 072350, Hebei, China; 3 Ya-jingZang, Department of Neurology, Tangxian people’s Hospital, Baoding 072350, Hebei, China

**Keywords:** Intravascular Interventional Therapy, Acute Ischemic Stroke, Cognitive Function, Cerebral Hemodynamics, Inflammatory Factors

## Abstract

**Objectives:**

To evaluate the clinical effect of intravascular interventional therapy in the treatment of acute ischemic stroke and its influence on cognitive function, cerebral hemodynamics and inflammatory factors as well as its clinical significance.

**Methods:**

Eighty patients with acute ischemic stroke admitted to Tangxian people’s Hospital from January 2018 to January 2020 were randomly divided into two groups. Patients in the control group were treated with conventional thrombolytic therapy on the basis of basic treatment, while patients in the experimental group were given intravascular interventional treatment on the basis of conventional treatment. Clinical effect, recovery of cognitive function and activities of daily living, improvement of cerebral hemodynamics indexes, and changes of inflammatory factors before and after treatment were analyzed by combining NIHSS score and symptom improvement before and two months after treatment, respectively.

**Results:**

The effective rate of the experimental group was 82.5% after treatment, and that of the control group was 60%, with a statistically significant difference (P =0.02). Mini-mental State Examination (MMSE) and Barthel scores of the experimental group were significantly improved compared with those of the control group after treatment, with statistically significant differences between the two groups (P=0.00). The mean cerebral vascular blood flow (Qm) of the experimental group increased significantly after treatment compared with that of the control group, while vascular characteristic impedance (ZCV) and peripheral resistance (Rv) decreased significantly in the experimental, with a statistically significant difference (ZCV, P=0.01; Rv, P=0.05); After treatment, TNF- A, CRP, IL-6 and other indicators in the experimental group were significantly lower than those in the control group, with statistically significant differences (P=0.00).

**Conclusions:**

Intravascular interventional therapy is an effective treatment regimen for acute ischemic stroke boasting a variety of advantages over conventional thrombolysis, such as significant symptom improvement, high efficacy, favourable recovery of cognitive function and activities of daily living, improvement of cerebral hsemodynamic indicators, and significant reduction of inflammatory cytokine levels.

## INTRODUCTION

Acute ischemic stroke, clinically common cerebral circulation disorder in middle-aged and elderly patients,^1^ has a pathogenesis of cerebral tissue infarction caused by cerebral artery occlusion. Currently, there is still a lack of specific treatment for this disease. It is the second leading cause of death in the world and a major cause of long-term disability, causing an annual global economic burden.^2^

It has been considered in relevant studies^3^ that restoring the effective blood supply of ischemic brain cells before the irreversible ischemic necrosis of brain cells plays an important role in restoring cerebral blood perfusion and improving the ischemic penumbra brain tissue.^4^ Intravenous thrombolytic therapy is currently the preferred treatment for this disease, and the commonly used drugs include recombinant tissue-type fibrinolytic activator (RT-PA) and urokinase. However, such a treatment method is limited in its clinical application owing to the narrow time window of intravenous thrombolysis, strict indications for medication and large side effects.^5^

In recent years, with the development of intravascular interventional therapy, mechanical stent interventional thrombus removal and intravascular thrombolysis have been gradually applied in clinical practice. This treatment method is widely sought after for its simple operation, quick operation, direct action on the lesion sites of blood vessels, and high vascular recanalization rate.^6^ Great progress has been made in the research on the mechanism of ischemic stroke. Studies have pointed out that^7^ inflammatory factors exert an important role in the formation of atherosclerotic plaques and can participate in reactions such as vascular endothelial injury and vascular restenosis. In this paper, intravascular interventional therapy was utilized to treat acute ischemic stroke.

## METHODS

A randomized controlled trial was used in this study. A total of 80 patients with acute ischemic stroke admitted to Tangxian people’s Hospital from January 2018 to January 2020 were selected and randomly divided into two groups according to random number table method: the control group and the experimental group, with 40 cases in each group. There were 22 males and 18 females in the experimental group, aged 54-73 years old, with an average of 66.11±4.84, while there were 24 males and 16 females in the control group, aged 51-74 years, with an average of 64.15±6.23. No significant difference was observed in the comparison of the general data between the two groups, which was comparable between the groups Table-I.

**Table I:** Comparative analysis of general data between the experimental group and the control group (χ¯±S) n=40.

Indicators	Experimental group	Control group	t/χ^2^	P
Age (years old)	66.11±4.84	64.15±6.23	1.57	0.12
Male (%)	22 (55%)	24 (60%)	0.20	0.65
**Infarct site**				
Internal carotid artery (%)	9 (22.5%)	11 (27.5%)	0.27	0.60
Middle cerebral artery M1 (%)	18 (45%)	17 (42.5%)	0.05	0.82
Middle cerebral artery M2 (%)	13 (32.5%)	10 (25%)	0.55	0.45
Education level				
Primary school	17 (42.5%)	19 (47.5%)	0.20	0.65
Middle school	10 (25%)	6 (15%)	1.25	0.26
University or above	13 (32.5%)	15 (37.5%)	0.22	0.63
**Medical history**				
Hypertension	30 (75%)	33 (82.5%)	0.67	0.41
Diabetes	26 (65%)	23 (57.5%)	0.47	0.49
Smoking history	29 (72.5%)	27 (67.5%)	0.24	0.62
History of alcoholism	13 (32.5%)	10 (25%)	0.55	0.45
NIHSS score	17.73±6.21	18.03±5.73	0.22	0.82
MMSE score	17.47±2.76	16.73±3.01	1.15	0.26
Barthel score	27.35±7.90	26.59±6.77	0.46	0.65

P>0.05.

### Ethical Approval:

The study was approved by the Institutional Ethics Committee of Tangxian people’s Hospital on March 10, 2018 (No.[2018]027), and written informed consent was obtained from all participants.

### Inclusion criteria:

- Patients who meet the diagnostic criteria of acute cerebral infarction^8^ by CT, MRI, digital subtraction angiography and other clinical examinations, and the duration of neurological symptoms is greater than one hour.
- Patients with onset time < 6h;
- Patients with moderate stroke or above (NIHSS score ≥ 5 points)^9^;
- Patients younger than 75 years old;
- Patients with moderate cognitive impairment (MMSE10-20 points)^10^;
- Patients with moderate to severe disorders in activities of daily living (Barthel index 20-60)^11^;
- Patients who themselves or their family members agree to the study and sign the consent form, and are able to cooperate with the study.

### Exclusion criteria:

- Patients with cerebral hemorrhage or subarachnoid hemorrhage confirmed by CT or MRI;
- Patients with severe heart, liver, and kidney insufficiency that cannot be satisfactorily controlled;
- Patients with recent (within three months) history of cerebral infarction, myocardial infarction or craniocerebral trauma or previous history of hemorrhagic disease;
- Patients with severe hypertension (Grade-III and above);
- Patients with severe abnormal blood coagulation function;
- Patients with incomplete clinical data.

Both groups were given basic treatment, such as lowering intracranial pressure, alleviating cerebral edema, scavenging oxygen free radicals, correcting water and electrolyte disorders, nutritional support, and nourishing brain cells, and other symptomatic treatments. Patients in the control group were treated with conventional thrombolytic therapy on the basis of basic treatment. The specific regimen was as follows: 10% of alteplase 0.9mg/kg was given intravenously within two minutes, and the remaining 90% was given intravenously by infusion pump within one hour, with the maximum dose not exceeding 100 mg. Butylphthalide 25mg was intravenously administered for >50 minutes, bid, with an interval of no less than 6h for consecutive 2 weeks.

Patients in the experimental group were given intravascular interventional treatment on the basis of conventional treatment: Patients were placed in supine position under local anesthesia. First, the right femoral artery of the patients was intubated and a 6 F catheter sheath was inserted. Then, the appropriate angiographic catheter was selected for whole-brain arteriography to understand the location and severity of embolization. For patients who had poor perforator angiography or no large artery occlusion, 100000 U urokinase were injected intravenously into the arterial trunk on the side with poor development or the vein on the opposite side of the lesion. Then, based on the development of the patients’ condition, urokinase were injected at a dose of 20000 U per minute for 15min. For patients with vascular occlusion, the catheter was guided by the micro-guide wire close to the position of the occluded blood vessel through the auxiliary role of the catheter, the stent was released and shaped, and the stent was observed under angiographic monitoring for 5min and then retracted. At the same time, the guide catheter was used to withdraw the broken thrombus, and 15mi later, angiography was performed again to observe and evaluate the patients’ vascular recanalization. In case of satisfactory recanalization, the procedure would be completed, otherwise the thrombectomy would be repeated. Head CT or MRI was re-examined 24hour postoperatively. If there was no obvious hemorrhage, aspirin was taken orally 100 mg at one tablet/daily.

### Observation Indicators:

*Observation of clinical effects:* Comprehensive analysis was conducted on the patients before treatment and 2 months after treatment by combining NIHSS score and symptom improvement.^12^ *Markedly effective:* Symptoms and signs have basically disappeared, and NIHSS score has improved by more than 90%; Effective: Symptoms and signs have significantly improved, and NIHSS score has improved by 45%-90%; Invalid: Symptoms and signs have not improved significantly, and NIHSS score has improved by less than 17%. Effective rate is equal to the sum of markedly effective and effective. *Recovery of cognitive function and activities of daily living:* MMSE scale and Barthel scale were used to evaluate the recovery of cognitive function and activities of daily living before treatment and 2 months after treatment. MMSE evaluation criteria: Normal: 27-30 points: mild cognitive impairment 21-27 points, moderate 10-20 points, severe 0-9 points; Barthel index evaluation criteria: The total Barthel index score is 100 points. Patients with a score of 60 or more have mild dysfunction, but can live on their own. Patients with a score of 40-60 have moderate dysfunction and need assistance in life. Patients with a score of 20-40 have severe dysfunction and need great help in life. Patients with a score of less than 20 are completely disabled and completely dependent on their lives. 3) Observation of cerebral hemodynamic indicators: Cerebral hemodynamic indexes, including mean cerebral vascular blood flow (Qm), vascular characteristic impedance (ZCV), and peripheral resistance (Rv), were detected by cerebrovascular function detector before treatment and 2 months after treatment, respectively, and the differences between the two groups were compared and analyzed. *Inflammatory factors:* 5ml of peripheral venous blood was drawn from all patients before treatment and in the morning after treatment, and enzyme-linked immunosorbent assay (ELISA) was used to detect tumor necrosis factor (TNF-a) and C-reactive protein (CRP), interleukin 6 (IL-6) and other inflammatory factors.

### Statistical Analysis:

All the data were statistically analyzed by SPSS 20.0 software, and the measurement data were expressed as (X̄± s). Two independent sample t-test was used for inter-group data analysis, paired t test was used for intra-group data analysis, and 2-test was adopted for rate comparison. P<0.05 indicates a statistically significant difference.

## RESULTS

Comparison of the two groups showed that the effective rate of the experimental group was 82.5%, which was significantly higher than that of the control group (60%), with a statistically significant difference (P=0.02, Table-II).

**Table II:** Comparative analysis of the clinical efficacy of the two groups (χ¯±S) n=40.

Group	Markedly effective	Effective	Invalid	Effective rate
Experimental group	14	19	7	33 (82.5%)
Control group	7	17	16	24 (60%)
χ^2^				4.94
P				0.02

p<0.05.

No statistically significant difference can be seen in the comparison of MMSE and Barthel scores between the experimental group and the control group before treatment (P > 0.05). Both MMSE and Barthel scores of the experimental group improved significantly compared with those of the control group after treatment, with a statistically significant difference was seen between the two groups (P=0.00, Table-III).

**Table III:** Comparative analysis of the recovery of cognitive function and activities of daily living in the two groups before and after treatment (χ¯±S) n=40.

Indicators		Experimental group	Control group	t	p
MMSE score	Before treatment	17.47±2.76	16.73±3.01	1.15	0.26
	After treatment*	25.74±3.79	22.06±2.21	5.30	0.00
Barthel score	Before treatment	27.35±7.90	26.59±6.77	0.46	0.65
	After treatment*	57.48±12.07	47.35±9.81	4.12	0.00

* p<0.05.

No statistically significant difference can be seen in the comparison between the experimental group and the control group in terms of mean cerebral vascular blood flow (Qm), vascular characteristic impedance (ZCV), peripheral resistance (Rv) and other indicators before treatment (P > 0.05). Qm of the experimental group increased significantly compared with the control group after treatment, with a statistically significant difference (P=0.00). ZCV and Rv were significantly lower in the experimental group than in the control group after treatment, with a statistically significant difference (ZCV, P=0.01; Rv, P=0.05) (Table-IV)

**Table IV:** Comparative analysis of cerebral hemodynamic indexes before and after treatment in the two groups (χ¯±S) n=40.

Indicators		Experimental group	Control group	t	p
Q_m_ (ml/s)	Before treatment	3.21±0.18	3.24±0.23	0.65	0.52
	After treatment*	4.65±0.73	4.24±0.55	2.84	0.00
ZCV (KP·s/m)	Before treatment	23.50±5.72	23.61±4.98	0.09	0.93
	After treatment*	17.63±3.38	20.64±3.72	3.78	0.01
Rv (KP·s/m)	Before treatment	76.75±11.27	75.73±10.98	0.41	0.68
	After treatment*	58.21±12.43	63.75±11.84	2.04	0.04

* p<0.05.

Again no statistically significant difference can be seen in the comparison of tnF-A, CRP, IL-6 and other indicators between the experimental group and the control group before treatment (P > 0.05). After treatment, tnF-A, CRP, IL-6 and other indicators of the experimental group were significantly reduced compared with the control group, showing a statistically significant difference (P=0.00) (Table-V).

**Table V:** Comparative analysis of changes in inflammatory factors before and after treatment in the two groups (χ¯±S) n=40.

Indicators		Experimental group	Control group	t	p
TNF-ɑ (ng/L)	Before treatment	45.36±11.25	45.62±11.7	0.68	0.21
	After treatment*	6.95±1.21	11.58±2.45	4.38	0.00
CRP (mg/L)	Before treatment	44.27±7.46	44.33±7.59	0.34	0.46
	After treatment*	6.42±1.13	10.79±3.07	11.27	0.00
IL-6 (ng/L)	Before treatment	10.26±1.84	10.35±1.73	0.27	0.64
	After treatment*	3.16±0.23	5.49±1.82	9.06	0.00

* p<0.05

## DISCUSSION

Acute ischemic stroke is characterized by high incidence, high disability rate and high mortality.^13^ Early reperfusion therapy can improve reversible brain cell damage, while thrombolytic therapy was once considered to be an effective means for the treatment of acute ischemic stroke, which can promote the recanalization of occluded cerebral arteries, quickly restore the blood flow of cerebral infarction lesions, and improve neurological function.^13^ Therefore, intravenous thrombolysis was once regarded as the preferred clinical treatment for acute ischemic stroke. However, intravenous thrombolysis also has certain shortcomings, such as low blood vessel recanalization rate, many complications, small safety time window, etc., coupled with individual differences in patients, making it difficult to achieve the desired results.^14^ It was considered in the study by Kumar et al.^15^ that patients with acute ischemic stroke were given intravenous thrombolysis at an ultra-early stage and the blood vessel recanalization was about 60%, and there are still some patients who are difficult to benefit from this treatment. In recent years, intravascular interventional therapy has attracted clinical attention, which includes intravascular thrombolysis and mechanical thrombectomy.^16^ The former can more effectively improve the local drug concentration, reduce the loss of thrombolytic drugs, and increase the contact area between thrombolytic drugs and emboli, so as to achieve the direct recanalization of blood vessels at the lesion site and quickly restore the oxygen supply of blood at the distal end of occluded vessels.^17^ Mechanical stent interventional thrombectomy boasts to support the diseased blood vessel, quickly remove the obstructed thrombus, restore and maintain smooth blood circulation,^18^ and can prolong the treatment time window, shorten the recanalization time, and improve the recanalization rate.

Previous studies have shown that intravascular intervention in acute stroke improved clinical outcomes without increasing overall mortality compared with intravenous thrombolysis.^19^ In our study, the effective rate of intravascular interventional therapy for acute ischemic stroke was 82.5%, and that of the control group was 60%, with a statistically significant difference (P=0.02), which was similar to the results of previous studies. The cerebral hemodynamics of the experimental group improved significantly after treatment, for example, Qm increased significantly compared with the control group, with a statistically significant difference (P=0.00), while ZCV and Rv decreased significantly in the experimental group compared with the control group, with a statistically significant difference (ZCV, P=0.01; Rv, P=0.05). It was confirmed in a randomized controlled trial by Silva et al.^20^ that the use of intravascular thrombectomy for mechanical thrombectomy for patients within 24h has still achieved favourable clinical results, and it was considered to have a time window of up to 8h for the anterior circulation and up to 24h for the posterior circulation. This can provide support for the results of our study.

In patients with ischemic stroke, severe microcirculation disturbances in the ischemic area lead to the massive generation of oxygen free radicals, resulting in an inflammatory waterfall effect, which further aggravates blood circulation disturbances and vascular pathological changes.^21^ Reperfusion may lead to the production of highly harmful reactive oxygen species (ROS) and produce oxidative stress (OS), which is the cause of most ischemia-reperfusion injury, leading to brain tissue damage.^22^ In our study, the MMSE score of cerebral cognitive function and Barthel score of activities of daily living were significantly improved compared with those of the control group (P=0.00). After treatment, TNF- A, CRP, IL-6 and other indicators in the experimental group were significantly lower than those in the control group, with statistically significant differences (P=0.00). It was considered in the study of Ospel et al.^23^ that intravascular interventional therapy is capable of significantly improving the ability of local brain cell inflammatory environment. Goktay et al.^24^ believed that intravascular interventional therapy could recanalize blood vessels faster, quickly reduce local inflammation, and reduce the risk of ischemia-reperfusion injury and rebleeding. These conclusions all support the viewpoint of our study.

### Limitations of this study:

A small number of cases are included, with short follow-up time. Moreover, only patients within six hour of the onset of the disease are included in the study in order to ensure the recovery of patients, and no comparative analysis was conducted between intravascular interventional therapy and conventional intravenous thrombolysis in patients within different time windows. In view of this, positive measures will be taken to accumulate cases, compare and analyze patients in different time windows, and analyze the specific schemes of intravascular interventional therapy, such as the comparative study of mechanical thrombectomy and intravascular thrombolysis, and follow-up time will continue to increase, so as to elaborate on the short-term and long-term effects of intravascular interventional therapy in more detail.

## CONCLUSION

Intravascular interventional therapy is an effective treatment regimen for acute ischemic stroke boasting a variety of advantages over conventional thrombolysis, such as significant symptom improvement, high efficacy, favourable recovery of cognitive function and activities of daily living, improvement of cerebral hemodynamic indicators, and significant reduction of inflammatory cytokine levels.

### Authors’ Contributions:

**KLL:** Designed this study and prepared this manuscript, and are responsible and accountable for the accuracy or integrity of the work.

**JH:** Collected and analyzed clinical data.

**YJZ:** Significantly revised this manuscript.
